# Development of a novel nomogram for predicting clinically significant prostate cancer with the prostate health index and multiparametric MRI

**DOI:** 10.3389/fonc.2022.1068893

**Published:** 2022-11-29

**Authors:** Li-Cai Mo, Xian-Jun Zhang, Hai-Hong Zheng, Xiao-peng Huang, Lin Zheng, Zhi-Rui Zhou, Jia-Jia Wang

**Affiliations:** ^1^Department of Urology, Taizhou Hospital of Zhejiang Province affiliated with Wenzhou Medical University, Linhai, Taizhou, Zhejiang, China; ^2^Department of Pathology, Taizhou Hospital of Zhejiang Province affiliated with Wenzhou Medical University, Linhai, Taizhou, Zhejiang, China; ^3^Department of Urology, Taizhou Cancer Hospital, Wenling, Taizhou, Zhejiang, China; ^4^Department of Radiation Oncology Center, Taizhou Cancer Hospital, Wenling, Taizhou, Zhejiang, China; ^5^Department of Radiation Oncology Center, Huashan Hospital, Shanghai Medical College, Fudan University, Shanghai, China; ^6^Department of Traditional Chinese Medicine, Taizhou Hospital of Zhejiang Province affiliated with Wenzhou Medical University, Linhai, Taizhou, Zhejiang, China

**Keywords:** prostate cancer, nomogram, multiparametric magnetic resonance imaging (mpMRI), prostate health index, predicting

## Abstract

**Introduction:**

On prostate biopsy, multiparametric magnetic resonance imaging (mpMRI) and the Prostate Health Index (PHI) have allowed prediction of clinically significant prostate cancer (csPCa).

**Methods:**

To predict the likelihood of csPCa, we created a nomogram based on a multivariate model that included PHI and mpMRI. We assessed 315 males who were scheduled for prostate biopsies.

**Results:**

We used the Prostate Imaging Reporting and Data System version 2 (PI-RADS V2) to assess mpMRI and optimize PHI testing prior to biopsy. Univariate analysis showed that csPCa may be identified by PHI with a cut-off value of 77.77, PHID with 2.36, and PI-RADS with 3 as the best threshold. Multivariable logistic models for predicting csPCa were developed using PI-RADS, free PSA (fPSA), PHI, and prostate volume. A multivariate model that included PI-RADS, fPSA, PHI, and prostate volume had the best accuracy (AUC: 0.882). Decision curve analysis (DCA), which was carried out to verify the nomogram’s clinical applicability, showed an ideal advantage (13.35% higher than the model that include PI-RADS only).

**Discussion:**

In conclusion, the nomogram based on PHI and mpMRI is a valuable tool for predicting csPCa while avoiding unnecessary biopsy as much as possible.

## Introduction

Prostate cancer (PCa) is the most frequent cancer among men for more than half of the globe, and it was the sixth major cause of death among men in 2020 (1). Although the frequency of PCa in China is lower than in European and American countries, it is rising at an alarming pace, which may have a negative impact on survival rate (2). Possible explanations include the rising incidence of PSA screening and the widespread use of multiparametric magnetic resonance imaging (mpMRI) in the clinic (3). PSA has been the most significant molecular biomarker for prostate cancer screening and postoperative follow-up since its discovery in the 1980s (4). However, the low specificity of PSA inevitably results in many needless biopsies, and detecting clinically insignificant prostate cancer is not desirable (5). Moreover, PSA testing mostly reveals indolent cancers that are unlikely to develop throughout a patient’s lifetime and that benefit from surgery or radiation only very infrequently (6).

In recent years, it has been shown that the Prostate Health Index (PHI), a mathematical formula that integrates total PSA (tPSA), free PSA (fPSA), and [-2] ProSA (p2PSA), is more effective than tPSA in the diagnosis of csPCa (7–9). The PHI blood test was authorized by the US Food and Drug Administration (FDA) in 2012 to detect PCa with elevated PSA (10). Prostate MRI is useful in diagnosing suspected prostate cancer and has a high negative predictive value (NPV) for csPCa (11). The Prostate Imaging Reporting and Data System, version 2 (PI-RADS v2), which was introduced recently, is a powerful tool to identify csPCa needing biopsy and help locate the lesions of the target (12). If the MRI is positive (PI-RADS score ≥3), biopsies should be conducted (13). However, PI-RADS 3 only identified csPCa in ≤20% of patients (14). In a meta-analysis, the positive predictive value(PPV) of mpMRI for csPCa was 40%, and the PPV in PI-RADS 4 and 5 lesions was still suboptimal (15). It is not advisable to use mpMRI alone to screen patients for biopsy (16). To the best of our knowledge, only a few studies have examined the predictive power of combining mpMRI with PHI in men with csPCa (17–19).

Therefore, we performed this study to conduct a novel nomogram incorporating PHI, mpMRI, and other variables to predict csPCa in a Chinese population.

## Materials and methods

### Patients

**Figure 1:**
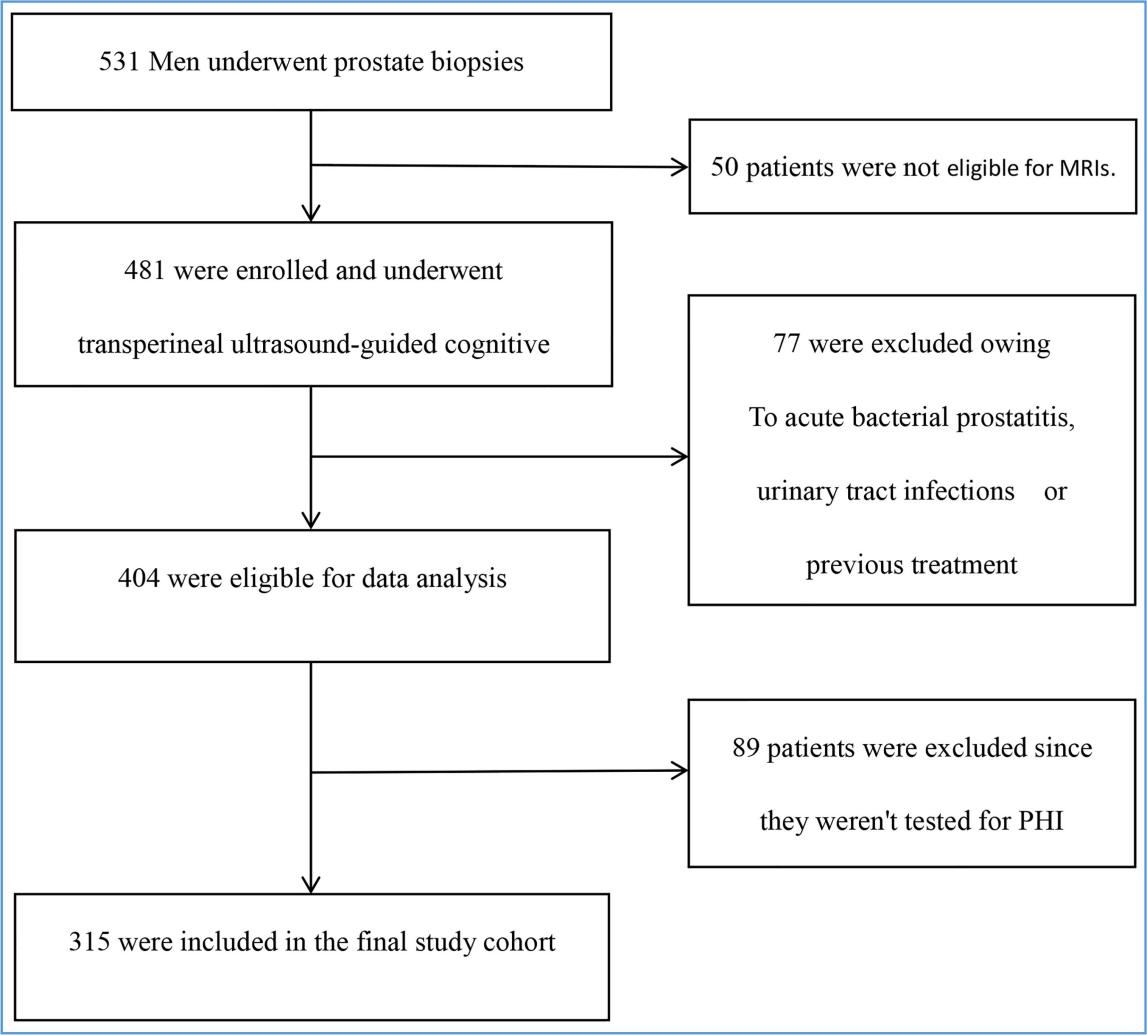
Enrollment and outcomes. The primary analysis included all 531 men. Those who were ineligible for MRIs or PHI, as well as those with acute bacterial prostatitis, urinary tract infections, or a history of treatment, were excluded. The final study cohort consisted of 315 people.

We performed a single-center study of patients with abnormal digital rectal examination (DRE) and/or increased blood tPSA who had biopsies between January 2020 and June 2022. Subjects with acute bacterial prostatitis, urinary tract infections, a history of PCa or a prostate biopsy, or who had taken any dose of 5-alpha reductase inhibitors were excluded. **Figure 1** illustrates the screening procedure.

### Biomarker measurement

The fPSA, tPSA, and p2PSA levels were determined prior to biopsy using the fully automated immunoassay equipment Access 2 analyzer (Beckman Coulter, Brea, CA, USA). PHI was calculated using the formula p2PSA/fPSA×√tPSA. The percentage of fPSA (%fPSA) was calculated as fPSA/PSA×100, while the percentage of p2PSA (%p2PSA) was defined as p2PSA/fPSA × 100. Moreover, PSA density (PSAD) and PHI density (PHID) were determined as the ratios of PSA/prostate volume and PHI/prostate volume, respectively.

### Multiparametric MRI

Without an intrarectal coil, mpMRI of the prostate was performed on a 3.0 T GE Signa HDx MR scanner (GE Healthcare, Milwaukee, USA) prior to biopsy. A senior abdominal radiologist with more than 8 years of expertise in prostate MRI and >300 scans yearly reviewed the mpMRI, which includes T2-weighted imaging (T2WI), diffusion-weighted imaging (DWI), b values of 0 and 800-1200 s/mm^2^, and apparent diffusion coefficient (ADC). The 2015 scoring rules for the PI-RADS V2 were used and a score between 1 and 5 was assigned (12).

### Biopsy protocol

Patients who had 3.0T mpMRI prior to biopsy received transperineal ultrasound-guided cognitive targeted biopsies for 2-3 cores based on lesions with PI-RADS(≥3) revealed on mpMRI as well as systematic biopsies for at least 12 cores using a biplane TRUS probe (Esaote, Transducer TRT33) and an 18-G disposable needle. Prostate volume was measured using TRUS during a monitoring biopsy. The ellipsoid formula (width × height × length × 0.52) was applied to calculate prostate volume.

### Histology

Tissue biopsies were examined by a single dedicated uropathologist. According to the 2014 consensus criteria from the International Society of Urological Pathology, PCa was evaluated as follows: grade group (GG) 1 (Gleason score ≤6), GG 2 (Gleason score 3 + 4 = 7), GG 3 (Gleason score 4 + 3 = 7), GG 4 (Gleason score 8), and GG 5 (Gleason score ≥9) (20). Clinically significant PCa was characterized in prostate biopsy pathology as GG2 ≥2, which is known to be more prevalent in the most recent definitions (21, 22).

### Ethics approval

The 1964 Declaration of Helsinki and its later amendments served as the ethical framework for this retrospective investigation. The local ethics commission decided not to require participants to provide their informed consent (Medical Ethics Committee of Taizhou Hospital, Zhejiang Province, China, K20220838).

### Statistical analysis

In the case of numerical variables, descriptive statistics (median (25th percentile; 75th percentile)) were utilized to define the entire sample. Percentages and absolute frequencies were used to represent categorical variables. We used Kolmogorov-Smirnov test to test the normal distribution of the continuity variables. For comparisons of continuous variables, the Mann-Whitney U test was utilized. To compare qualitative variables, a chi-squared test was used. We performed ROC analysis to compare the diagnostic accuracy of mpMRI, individual PSA-derived blood indicators, and a combination of the two, and we determined the area under the curve (AUC) and its 95% confidence interval. The DeLong test was employed to evaluate the AUCs of the various prediction models (23). The baseline characteristics of patients (prostate volume), laboratory data (PHI, fPSA), and PI-RADS category were used to develop multivariable logistic models for predicting csPCa. Using a nomogram, the best prediction model was presented for clinical use. Bootstrap resampling (1000 repeats) was utilized to examine both discrimination and calibration for internal model validation. To assess predictive accuracy, a bootstrap method was utilized to obtain bootstrap-corrected estimates of the C-Statistics. Visual and statistical calibration were examined using the Hosmer-Lemeshow test. The Youden index was maximized to obtain an ideal threshold (sensitivity + specificity - 1). IBM SPSS 25.0 (IBM Corp., Armonk, NY, USA) was used for statistical analyses. The results were judged to be statistically significant when the *p*-value was <0.05. Using R and the rms and rmda packages, we were able to visualize nomograms, decision curves, and calibration curves (version. 4.2.1, R Foundation for Statistical Computing, Vienna, Austria).

## Results

### Clinical characteristics of the study cohort

The clinical characteristics of the patients and the results of auxiliary examination are shown in **Table 1**. Overall, in the diagnostic setting, 194 patients (61.6%) had a positive biopsy. Patients with negative biopsy and those with PCa on biopsy were compared in terms of age, PI-RADS score, and all PSA-derived serum indicators. The median fPSA concentration was comparable between groups (*P* = 0.915). Moreover, %fPSA was lower (12.04 vs. 15.90; *P <*0.001), while p2PSA, %p2PSA, PHI, PSAD and PHID were higher (42.68 vs. 24.76; 2.91 vs. 1.01; 28.23 vs. 17.33; 96.99 vs. 53.22; 0.29 vs. 0.17; 2.91 vs. 1.01, *P* < 0.001) in patients with PCa.

**Table 1:** Demographic and clinical characteristics of the study population.

	Overall	Negative biopsy	PCa	
Parameter	(n = 315)	(n = 121)	(n = 194)	*P* value
Age (median [IQR]), years	69.00 [64.00, 73.00]	68.00 [61.00, 73.00]	69.00 [65.00, 73.00]	0.118
MRI PI-RADS (%)				<0.001
1	10 (3.2)	8 (6.6)	2 (1.0)	
2	94 (29.8)	57 (47.1)	37 (19.1)	
3	97 (30.8)	36 (29.8)	61 (31.4)	
4	77 (24.4)	17 (14.0)	60 (30.9)	
5	37 (11.7)	3 (2.5)	34 (17.5)	
Prostate volume (median [IQR]), mL	36.04 [26.63, 56.58]	50.40 [32.55, 73.63]	32.94 [24.36, 43.08]	<0.001
tPSA (median [IQR]), ng/mL	9.97 [6.09, 16.54]	8.66 [5.03, 14.26]	10.71 [7.04, 18.97]	0.001
fPSA (median [IQR]), ng/mL	1.42 [0.85, 2.31]	1.43 [0.89, 2.13]	1.39 [0.84, 2.43]	0.915
p2PSA (median [IQR]), pg/mL	36.06 [21.31, 57.14]	24.76 [13.04, 40.80]	42.68 [26.66, 67.74]	<0.001
%fPSA (median [IQR])	13.36 [9.62, 19.22]	15.90 [10.75, 22.72]	12.04 [9.04, 16.73]	<0.001
%p2PSA (median [IQR])	25.02 [16.54, 35.80]	17.33 [12.31, 26.14]	28.23 [20.98, 39.05]	<0.001
PHI (median [IQR])	78.40 [52.33, 118.11]	53.22 [35.86, 76.63]	96.99 [70.12, 150.43]	<0.001
PSAD (median [IQR]), ng/mL/mL	0.24 [0.15, 0.51]	0.17 [0.10, 0.28]	0.29 [0.19, 0.66]	<0.001
PHID (median [IQR])	2.09 [1.03, 3.63]	1.01 [0.70, 1.79]	2.91 [1.85, 4.80]	<0.001

PI-RADS, Prostate Imaging Reporting and Data System; tPSA, total prostate specific antigen; p2PSA, [−2]proPSA; %fPSA,free PSA/total PSA;%p2PSA, p2PSA/free PSA; PHI, prostate health index; PSAD, PSA density, PSA/Prostate volume; PHID, PHI density, PHI/Prostate volume.

Of these, 158 (50.2%) had csPCa on biopsy (**Table 2**). To evaluate csPCa’s capacity to distinguish between patients, ROC analyses were carried out. Both the PI-RADS score and PSA-derived biomarkers were substantially associated with the likelihood of csPCa in univariable logistic analysis, with an AUC ranging from 0.52 (95% CI: 0.46 to 0.59) for free PSA to 0.83 (95% CI: 0.79 to 0.88) for PHID. As demonstrated in **Table 2**, PHID surpassed both the PI-RADS score (AUC: 0.75 (95% CI: 0.70 to 0.81)) and the tPSA (AUC: 0.63 (95% CI: 0.57 to 0.69)) in terms of diagnostic accuracy. In terms of PHI, the optimal cutoff was 77.77, with a corresponding sensitivity of 0.76 (95% CI: 0.68 to 0.82) and specificity of 0.73 (95% CI: 0.65 to 0.80). With a sensitivity of 0.70 (95% CI: 0.62 to 0.77) and specificity of 0.83 (95% CI: 0.76 to 0.89), the optimum threshold for PHID was found to be 2.36. The best cut-off value was established to be a PI-RADS score of 3, with a sensitivity of 0.85 (95% CI: 0.79 to 0.90) and a specificity of 0.52 (95% CI: 0.44 to 0.60) (**Table 2**).

**Table 2:** AUC comparing patients with clinically significant prostate cancer (csPCa) versus patients with negative biopsy or clinically insignificant PCa (No csPCa).

	No csPCa	csPCa					
Studied Variables	(n = 157)	(n = 158)	*P* value	AUC (95% CI)	Cut-off	Sensitivity	Specificity
Age (median [IQR]), years	68.00 [63.00, 73.00]	70.00 [65.00, 73.00]	0.147	0.55 (0.48-0.61)	69.5	0.51	0.60
MRI PI-RADS (%)			<0.001	0.75 (0.70-0.81)	3	0.85	0.52
1	9 (5.7)	1 (0.6)					
2	72 (45.9)	22 (13.9)					
3	47 (29.9)	50 (31.6)					
4	25 (15.9)	52 (32.9)					
5	4 (2.5)	33 (20.9)					
Prostate volume (median [IQR]), mL	45.11 [30.28, 71.98]	32.64 [23.71, 40.46]	<0.001	0.69 (0.63-0.75)	17.66	0.98	0.03
tPSA (median [IQR]), ng/mL	8.70 [5.19, 14.06]	10.91 [7.28, 21.22]	<0.001	0.63 (0.57-0.69)	17.57	0.32	0.87
fPSA (median [IQR]), ng/mL	1.42 [0.87, 2.13]	1.42 [0.84, 2.52]	0.521	0.52 (0.46-0.59)	2.02	0.37	0.75
p2PSA (median [IQR]), pg/mL	26.81 [14.55, 42.68]	46.16 [29.27, 70.82]	<0.001	0.71 (0.66-0.77)	35.26	0.68	0.66
%fPSA (median [IQR])	15.85 [10.75, 22.16]	11.66 [8.72, 15.21]	<0.001	0.67 (0.61-0.73)	50.03	0.02	1
%p2PSA (median [IQR])	19.58 [12.69, 28.67]	29.72 [22.43, 41.09]	<0.001	0.71 (0.65-0.77)	22.29	0.76	0.62
PHI (median [IQR])	55.79 [38.29, 80.16]	100.20 [78.23, 156.05]	<0.001	0.81 (0.75-0.85)	77.77	0.76	0.73
PSAD (median [IQR]), ng/mL/mL	0.18 [0.11, 0.29]	0.34 [0.21, 0.75]	<0.001	0.74 (0.68-0.79)	0.26	0.65	0.71
PHID (median [IQR])	1.13 [0.77, 2.10]	3.36 [2.09, 5.25]	<0.001	0.83 (0.79-0.88)	2.36	0.70	0.83
PI-RADS + volume			<0.001	0.80 (0.75-0.85)		0.81	0.71
PI-RADS+ PHI			<0.001	0.85 (0.81-0.89)		0.79	0.81
PI-RADS + volume+PHI			<0.001	0.87 (0.84-0.91)		0.81	0.82
PI-RADS+volume +PHI+ fPSA			<0.001	0.88 (0.85-0.92)		0.83	0.83

The AUC of the matching ROC curve with the 95% confidence interval (CI) is provided for each variable. According to the Youden index maximization, the best threshold for the diagnosis of PCa is provided with the achieved sensitivity and specificity. Cases are defined as individuals with values equal to or more than the threshold, with the exception of prostate volume, which is the inverse. PI-RADS, Prostate Imaging Reporting and Data System; tPSA, total prostate specific antigen; p2PSA, [−2]proPSA; %fPSA,free PSA/total PSA;%p2PSA, p2PSA/free PSA; PHI, prostate health index; PSAD, PSA density, PSA/Prostate volume; PHID, PHI density, PHI/Prostate volume; AUC, area under curve; ROC, receiver operating characteristic.

### Correlation between PHID and PI-RADS score

**Figure 2:**
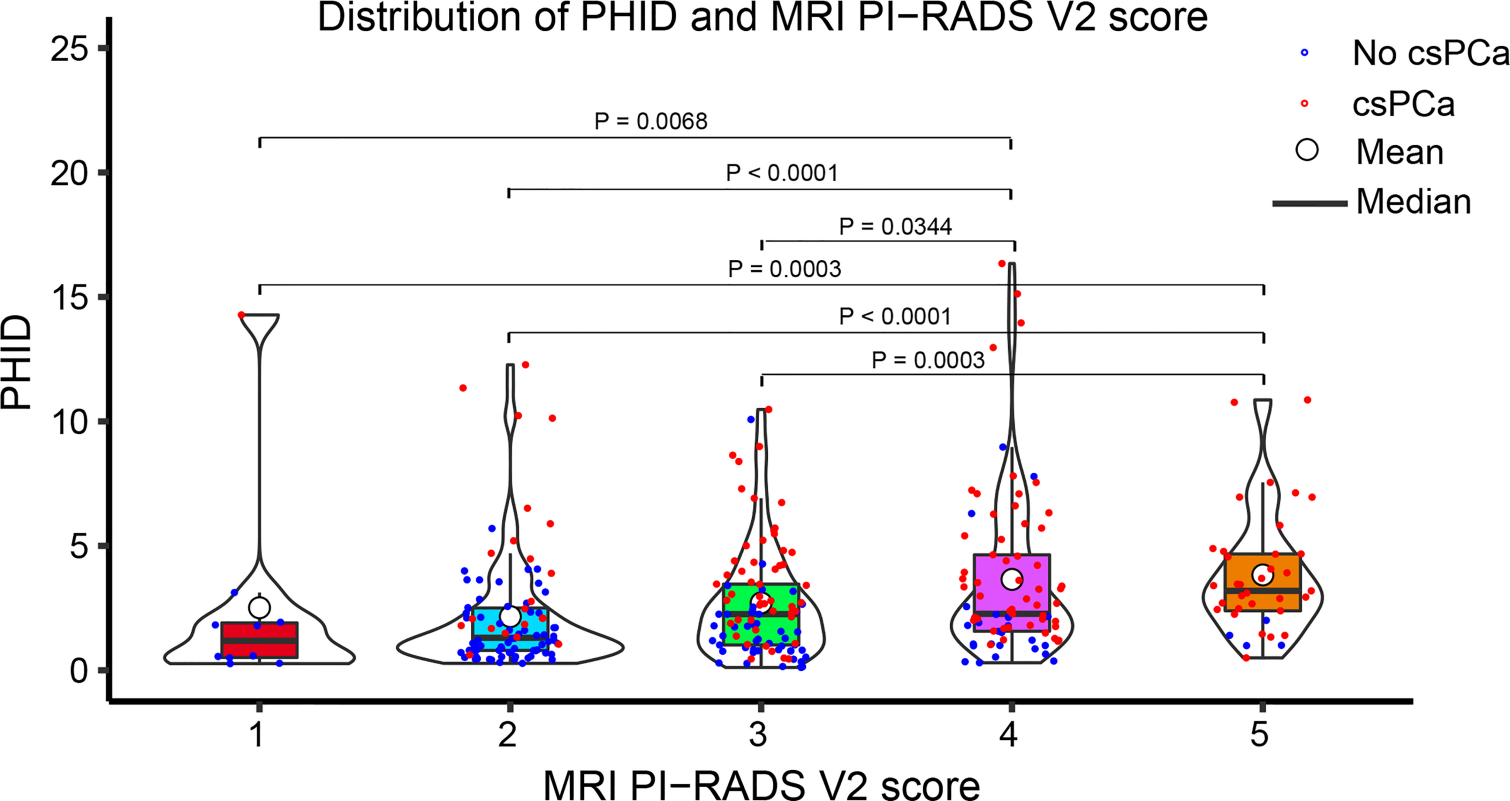
Violin plot showing the distribution of PHID according to the PI-RADS v2 score. Data are shown as the median (bold horizontal line in the box) and Q1 and Q3 (borders of the box). *Q1 = 25th percentile; Q3 = 75th percentile; IQR (interquartile range) = Q3-Q1.*.

The distribution of PHID and prostate biopsy pathology findings classified by PI-RADS score grade is shown in **Figure 2**. Patients with PI-RADS 4 presented an increased likelihood of harboring csPCa compared with those with PI-RADS 1 (OR 18.72 (95% CI: 2.25-156.01); *P* = 0.0068), PI-RADS 2 (OR 6.81 (95% CI: 3.47-13.37); *P* < 0.0001), and PI-RADS 3 (OR 1.96 (95% CI: 1.05-3.64); *P* = 0.0344). Patients with PI-RADS 5 presented an increased likelihood of harboring csPCa compared with those with PI-RADS 1 (OR 74.25 (95% CI: 7.36-749.46); *P* = 0.0003), PI-RADS 2 (OR 27.00 (95% CI: 8.62-84.61); *P* < 0.0001), and PI-RADS 3 (OR 7.75 (95% CI: 2.55-23.57); *P* = 0.0003).

### Nomogram development and internal validation for predicting csPCa

**Figure 3:**
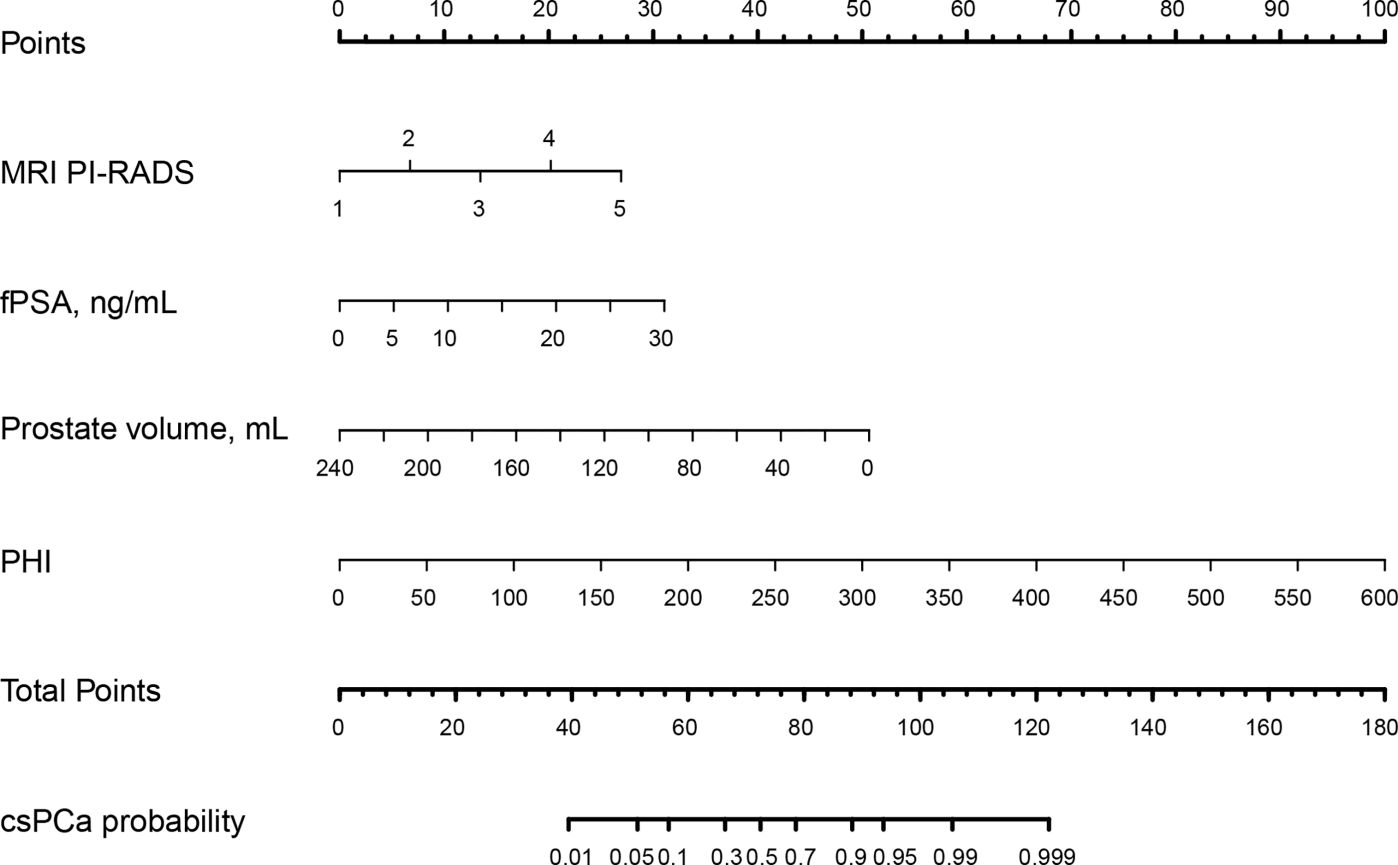
The nomogram was employed by determining the patient’s location on each factor axis. The scores for each element are summed to obtain a total score, which is depicted on the lower axis and corresponds to the likelihood of csPCa. *PI-RADS, Prostate Imaging Reporting and Data System; fPSA: free PSA; PHI: prostate health index; tPSA: total PSA; csPCa: clinically significant prostate cancer.*.

**Figure 4:**
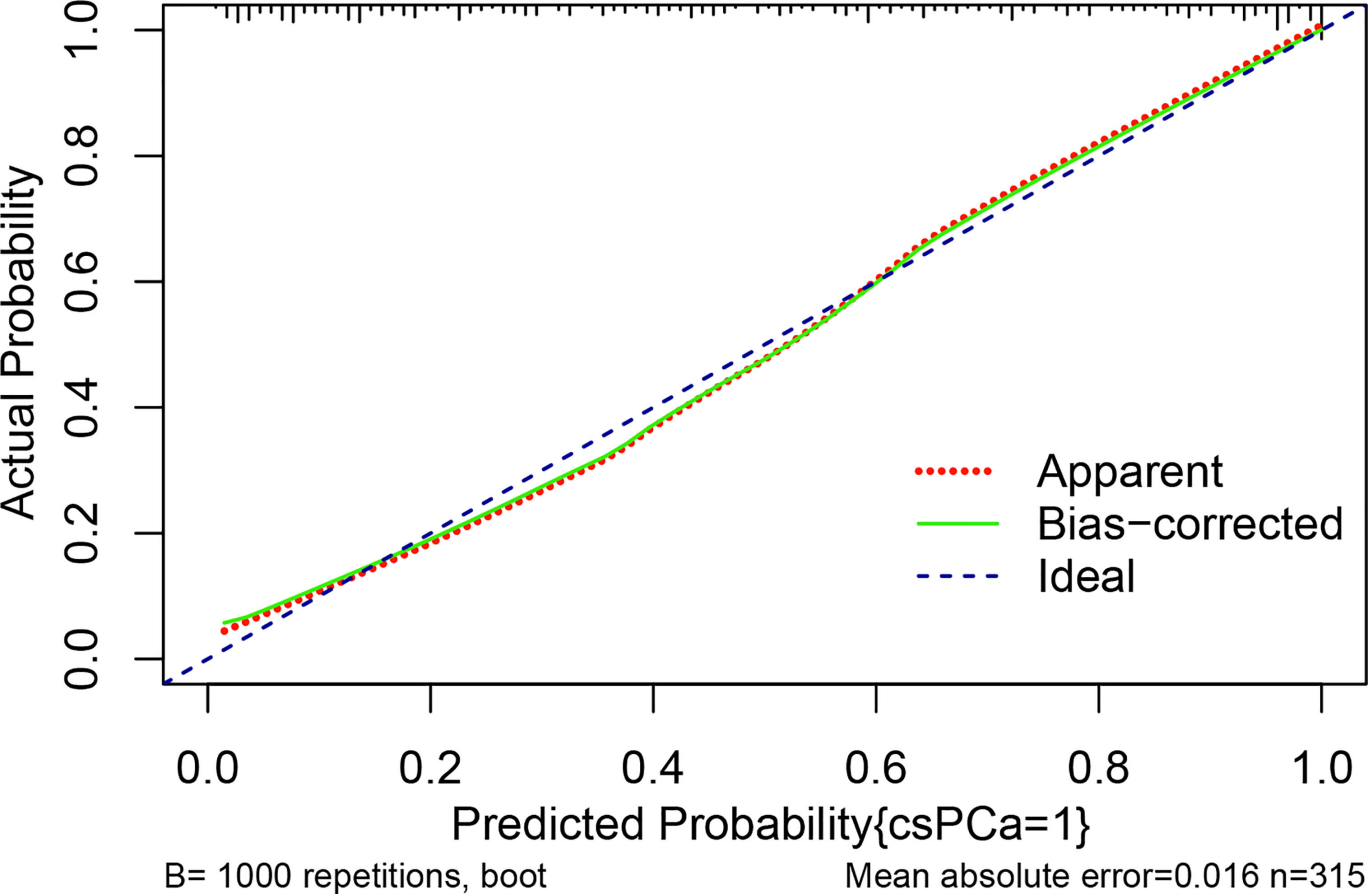
The calibration curve compares the accuracy of the original prediction model (“apparent”) (light dashed red line) to the bootstrap model (“bias corrected”) (solid green line) in predicting the likelihood of csPCa. The ideal calibration is shown by the diagonal blue dot line, which serves as the reference line. The abscissa indicates the anticipated value determined from the nomogram, whereas the ordinate reflects the observed actual probability value. Small vertical lines at the top of the graph represent the distribution of the expected probability. Overprediction occurs when an estimate falls below the reference line, while underprediction occurs when an estimate rises above the reference line. In an ideal situation, these two lines should be close to the diagonally dashed blue line.

**Figure 5:**
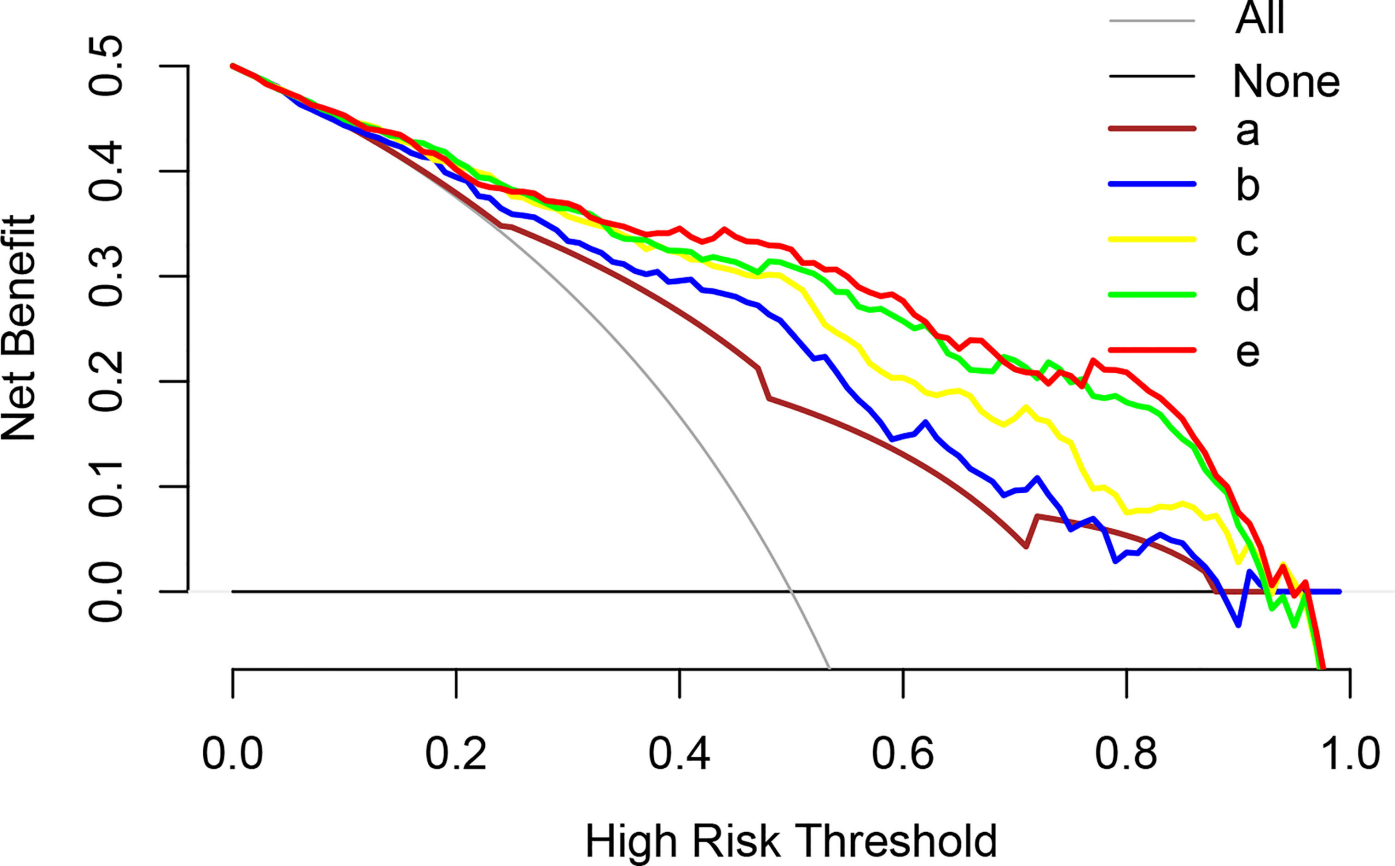
Analysis of the decision curves of several basic models and nomogram models. a: MRI PI-RADS; b: MRI PI-RADS + volume; c: MRI PI-RADS + PHI; d: MRI PI-RADS + volume + PHI; e: MRI PI-RADS + volume + PHI + fPSA (Nomogram model). *PI-RADS, Prostate Imaging Reporting and Data System; tPSA, total prostate-specific antigen; PHI, prostate health index.*.

Multivariable logistic analysis using PI-RADS, fPSA, PHI, and prostate volume yielded an AUC of 0.882 (95% CI: 0.845 to 0.920), and a nomogram was created to display these findings visually (**Table 2**, **Table 3**, and **Figure 3**). The PHI and mpMRI nomograms were internally tested using a 1000 bootstrap resampled dataset. The computed bootstrap-corrected C-index of 0.877 demonstrates excellent discriminative power. According to the calibration plot, there was a high degree of agreement between the calculated and observed probabilities (**Figure 4**). As measured by the Hosmer-Lemeshow goodness-of-fit test, the model was calibrated well (*P* = 0.829). Decision curve analysis (DCA) was carried out to verify the nomogram’s clinical applicability, and the results showed a maximum advantage (13.35% higher than the model that include PI-RADS only) (**Figure 5**). Furthermore, as shown in **Figure 5**, the net clinical benefit was 0.3206 based on the 50% likelihood threshold of what was expected.

**Table 3:** Multivariate logistic regression analysis of predictors in the prediction of csPCa.

Predictor	Odds Ratio	95% CI.	coefficient	*P* value
MRI PI-RADS	6.50	3.43-12.33	1.87	<0.0001
Prostate volume	0.42	0.27-0.63	-0.88	<0.0001
PHI	4.59	2.83-7.44	1.52	<0.0001
fPSA	1.23	1.04-1.46	0.21	0.0164

PI-RADS, Prostate Imaging Reporting and Data System; csPCa, clinically significant prostate cancer; PHI, prostate health index.

## Discussion

To our knowledge, this is a novel viable prediction nomogram model based on PHI and mpMRI that may enhance csPCa detection, greatly lowering PCa overdiagnosis prior to prostate biopsy in an Asian population. The net clinical benefit in this research was 0.3206, indicating that the nomogram would increase the identification of csPCa by 32.06%, leading to fewer needless biopsies when compared to a threshold possibility of 50%.

A change in cancer diagnostic priorities from detecting all cancers to focusing on the identification of potentially aggressive but curable cancers and minimizing the detection and treatment of indolent disease has resulted from the recognition of PCa overdiagnosis and overtreatment (24). In recent years, numerous novel biomarkers, including PHI, have shown promise in distinguishing clinically significant from inconsequential PCa more reliably than PSA (25–27). Extensive multicenter research has shown that employing a PHI threshold of 24, could have prevented 36%–41% of needless biopsies and 17%-24% of overdiagnosed indolent cancers (28). Additionally, the PHI has been verified in Asian populations. According to the results of a multicenter study conducted in China, Na et al. revealed that using a PHI of ≤35 as the threshold would have resulted in the missed diagnosis of 8% of PCa in prostate biopsies and 4% of high-grade lesions (29). The efficacy of PHI was analyzed across many Asian populations in a multicenter study. Using PHI criteria of 25, 25-35, 35-55, and >55, higher-grade PCa (Gleason 7) was found to be diagnosed in 1.0%, 1.9%, 13%, and 30% of Asian males, respectively (30).

The success of PSAD encouraged several authors to assess the use of PHID, which was originally reported in 2014 by Mearini et al. (31). The comparison between PHID and PHI showed equivocal findings. In a large cohort of naïve biopsy patients in whom PHI and PHID were compared, PHI seemed to perform better than PHID, especially in those with small prostates (under 40 cc) (32). Additionally, Garrido et al., in a cohort of patients with tPSA less than 10 ng/ml, revealed that PHID had a better diagnostic performance than PHI for overall PCa detection, not specifically for csPCa (33). However, in contrast to these findings, Tosoian et al. observed a considerably higher AUC for PHID (0.84 vs. 0.76 for PHI) in csPCa, leading to future research investigating its value (34). More recently, Druskin et al. reported a higher AUC for PHID (0.82) versus PHI (0.79) in the diagnosis of csPCa on biopsy (35). Our results also showed that PHID performed better than PHI, with AUCs of 0.83 and 0.81, respectively. Finally, Chiu et al. found that combining PHI and prostate volume (as PHID) is an excellent indicator of csPCa with an AUC of 0.82. For csPCa, PHID was most effective in preventing unnecessary biopsies (43.7%), achieving 90% sensitivity, and missing the fewest cases (8.5%) when PHID was ≥0.67 (25).

However, these studies did not include prostate MRI, a crucial technology that is increasingly being used in PCa diagnosis. The American Urological Association (AUA) concurs that lesions discovered on prostate mpMRI with PI-RADS ≥3 should be biopsied immediately and that biopsy of PI-RADS 3 lesions should not typically be postponed (36). The PPV of prostate mpMRI, however, is not optimal. Vendrink et al. discovered that 17%, 34%, and 67% of patients with PI-RADS 3, 4, and 5 lesions were diagnosed with csPCa, respectively (37). Together, PHI and MRI have been shown to improve diagnostic performance, allowing doctors to forego needle biopsies while still detecting the presence of advanced PCa. Hsieh et al. demonstrated in a prospective Asian cohort study that if prostate biopsies were only conducted in individuals with PI-RADS ≥3 and PHI ≥30 and just 1 patient with csPCa was excluded, roughly half of these biopsies could be avoided (22). Druskin et al. showed in a study of 104 men that the PI-RADS scores were complimentary to PHID and that the great majority of csPCa patients were diagnosed with a PI-RADS score of 1 or 2, a PHID ≥0.44 or, if a PI-RADS score of 3, 4, or 5 (35). These data confirm our hypothesis that a unique nomogram based on PHID/PHI and multiparametric MRI might be a valuable tool for predicting csPCa.

To our knowledge, this is a novel feasible predictive nomogram model based on PHI and mpMRI for assessing the PHI and PI-RADS scoring systems as a method of avoiding needless needle biopsies and combating overdiagnosis. As a result, the integration of PHI and mpMRI shows potential for csPCa assessment prior to prostate biopsy, which might improve patient quality of life and save healthcare costs. In the current study, we created and internally verified a novel nomogram based on PHI and MRI, achieving excellent csPCa detection accuracy (AUC 0.882). In addition, when compared to the other examined models, the highest net advantage for csPCa detection was achieved with a substantial cutoff possibility. In addition, the net clinical benefit was 0.3206 based on the 50% likelihood threshold of what was projected to happen, indicating that the nomogram would increase the detection of csPCa by 32.06%, hence lowering the number of needless biopsies.

Our results must be evaluated against some constraints. First, over the past few years, csPCa has been defined in a variety of different ways, and there is still no universally agreed upon definition. Overall, the most recent criteria seem to favor a Gleason score of >6 (ISUP >1); hence, we utilized that to designate csPCa in our analysis (38). Second, the analysis was based on prostate biopsy pathology, which is a drawback of this study. Because some individuals in this group who were diagnosed with prostate cancer selected brachytherapy or radiotherapy, the postoperative pathology of prostate cancer could not be collected, and csPCa may have been underestimated. The pathological upgrading rates of systematic biopsy alone, targeted biopsy alone, and targeted biopsy paired with systematic biopsy were 16.8%, 8.2%, and 3.5%, respectively (39, 40). Furthermore, in our investigation, targeted biopsy was performed using cognitive registration, which may be less accurate than MR/US fusion systems.

In conclusion, our PHI- and mpMRI-based nomograms are good prediction tools that may enhance the identification of patients with csPCa while avoiding unnecessary biopsies as much as possible. However, further research is required to externally confirm this nomogram and enhance csPCa detection.

## Data availability statement

The original contributions presented in the study are included in the article/Supplementary Material. Further inquiries can be directed to the corresponding authors.

## Ethics statement

The studies involving human participants were reviewed and approved by Medical Ethics Committee of Taizhou Hospital, Zhejiang Province, China, K20220838. Written informed consent for participation was not required for this study in accordance with the national legislation and the institutional requirements.

## Author contributions

LZ, Z-RZ, J-JW, and L-CM conceived and designed the study and drafted the manuscript. L-CM, X-JZ, H-HZ, and X-PH collected, analyzed, and interpreted the data. LZ, Z-RZ, and J-JW participated in revising the manuscript.

## Funding

This work was supported by the National Natural Science Foundation of China (grant 82003231 to Dr. ZRZ), the Traditional Chinese Medicine Science and Technology Plan Project of Zhejiang Province (No. 2022ZB388), Scientific Research Fund of Taizhou Enze Medical Center (Group) (No. 15EZD36), and the Wenling Science and Technology Program (No. 2020S0040001, 2021S00081, and 2021S00088).

## Conflict of interest

The authors declare that the research was conducted in the absence of any commercial or financial relationships that could be construed as a potential conflict of interest.

## Publisher’s note

All claims expressed in this article are solely those of the authors and do not necessarily represent those of their affiliated organizations, or those of the publisher, the editors and the reviewers. Any product that may be evaluated in this article, or claim that may be made by its manufacturer, is not guaranteed or endorsed by the publisher.
